# Chemical Composition and Biological Activities of Bulgarian Thyme (*Thymus callieri* Borbás ex Velen) and Summer Savory (*Satureja hortensis* L.) Essential Oils

**DOI:** 10.3390/cimb48050470

**Published:** 2026-05-01

**Authors:** Yulian Tumbarski, Ivan Ivanov, Ivayla Dincheva, Albena Parzhanova, Mina Pencheva

**Affiliations:** 1 Department of Microbiology and Biotechnology, University of Food Technologies, 26 Maritsa Blvd., 4002 Plovdiv, Bulgaria; 2 Department of Organic Chemistry and Inorganic Chemistry, University of Food Technologies, 4002 Plovdiv, Bulgaria; ivanov_ivan.1979@yahoo.com; 3 Department of Agrobiotechnologies, Agrobioinstitute, Agricultural Academy, 1164 Sofia, Bulgaria; ivadincheva@yahoo.com; 4 Department of Food Technologies, Institute of Food Preservation and Quality, Agricultural Academy, 4002 Plovdiv, Bulgaria; albenadsp@abv.bg; 5 Department of Medical Physics and Biophysics, Medical University of Plovdiv, 4002 Plovdiv, Bulgaria; mina.pencheva@mu-plovdiv.bg

**Keywords:** essential oils, *Thymus callieri*, *Satureja hortensis*, antioxidant activity, antimicrobial activity, anti-inflammatory activity, antihemolytic activity

## Abstract

Thyme (*Thymus callieri* Borbás ex Velen) and summer savory (*Satureja hortensis* L.) are aromatic plants from the Lamiaceae family widely used in traditional medicine and the food industry. This study provides a comparative analysis of the phytochemical profiles and biological potential of the essential oils (EOs) of these two plant species from Bulgaria. The chemical composition was determined using GC-MS analysis. Biological evaluation included determination of antioxidant activity (DPPH assay), antimicrobial activity (MIC assay), ex vivo anti-inflammatory effects (IL-1β expression in rat stomach smooth muscle preparations), and in vitro antihemolytic activity. GC-MS analysis identified 16 compounds in *T. callieri* EO, dominated by *p*-cymene (46.42%) and thymol (35.80%). In contrast, 17 compounds were identified in *S. hortensis* EO, with carvacrol (58.81%) and γ-terpinene (22.46%) as major constituents. Both EOs exhibited concentration-dependent antioxidant activity, with *S. hortensis* showing higher radical scavenging potential. In antimicrobial tests, both oils demonstrated broad-spectrum efficacy with MIC values ranging from 0.313 to 2.5 mg/mL. Ex vivo experiments revealed that *T. callieri* EO significantly increased IL-1β expression, suggesting immune activation, while *S. hortensis* EO showed a lower effect, indicating higher anti-inflammatory potential. Furthermore, *S. hortensis* EO demonstrated superior erythrocyte membrane stabilization (antihemolytic activity) compared to *T. callieri* EO and the reference anti-inflammatory drug Aspirin. In conclusion, the findings highlighted the distinct biological potential of both Bulgarian EOs, suggesting their diverse applicability as natural bioactive agents in the pharmaceutical and food industries.

## 1. Introduction

Essential oils (EOs) are natural, hydrophobic, highly aromatic liquids extracted from various parts of plants, including leaves, flowers, seeds, and peels, typically through distillation or cold pressing. These complex mixtures contain a wide range of secondary metabolites, including terpenes, alcohols, aldehydes, phenolic compounds, and other volatile compounds, which are responsible for their distinctive chemical and biological properties. In recent decades, scientific interest in EOs has increased substantially due to their diverse bioactivities, including antimicrobial, antioxidant, anti-inflammatory, immunomodulatory, and neuromodulatory effects. In parallel, EOs are increasingly applied in alternative medicine, the fragrance industry, and food systems, where they contribute to flavor enhancement, biopreservation, and overall product quality [1,2].

Recent studies have highlighted the antimicrobial potential of EOs and their components against a broad spectrum of microorganisms, including Gram-positive and Gram-negative bacteria, yeasts, and pathogenic fungi. EOs exert their bacteriostatic and bactericidal effects primarily by disrupting microbial cell membranes and inhibiting biofilm formation. These mechanisms support the potential use of EOs as natural alternatives or adjuncts to conventional antimicrobial agents, particularly in the context of rising resistance to traditional antibiotics [2]. In addition to antimicrobial activity, EOs have been shown to possess antioxidant and anti-inflammatory properties. Radical scavenging assays have indicated that various EOs can neutralize reactive oxygen species (ROS), suggesting their potential in mitigating oxidative stress. Certain EOs components, such as eugenol, thymol, cinnamaldehyde, and carvacrol, have been shown to modulate some key inflammatory signaling pathways, thereby reducing pro-inflammatory cytokine expression [3]. Recent reports revealed that EOs can influence neurological functions, including emotional regulation, cognitive performance, stress response, and autonomic nervous system activity, potentially via olfactory and respiratory pathways. In addition to their direct cellular effects, EOs may also interact with the central nervous system, supporting their traditional use in aromatherapy to alleviate pathophysiological conditions (pain, depression, stress, anxiety, sleep disturbances, agitated behavior, and mental fatigue) [4].

Thyme is a widely distributed perennial aromatic plant belonging to the Lamiaceae family. The *Thymus* genus encompasses approximately 250 taxa, comprising 214 species and 36 subspecies, native to Europe (mainly the Mediterranean region), the Middle East, and North Africa. Among the *Thymus* species, *Thymus vulgaris* L. (common thyme) is the most commonly cultivated for culinary, medicinal, and ornamental purposes. In Bulgaria, 21 *Thymus* species have been described, including *Thymus callieri* Borbás ex Velen, which has been observed near the town of Dospat in the Rhodope Mountains at an altitude of approximately 1200 m, among other locations [5,6].

Phytochemical analyses indicate that species of the *Thymus* genus contain phenolic acids, flavonoids, alkaloids, tannins, and saponins as non-volatile constituents, in addition to essential oil as a volatile component. The essential oil obtained from the aerial parts of the plant is composed predominantly of terpenes, including thymol, carvacrol, borneol, linalool, geraniol, and sabinene hydrate, which have been reported to exhibit antimicrobial, antioxidant, anti-inflammatory, antispasmodic, bronchodilatory, and other pharmacological activities [5]. Based on the relative proportions of these major constituents, several chemotypes of *Thymus* ssp. essential oil can be distinguished, which are influenced by species, geographical origin, and environmental conditions [7]. Recent studies on *Thymus callieri* from Struma River Valley, Bulgaria, have demonstrated that EO yield was 2.11% and that it contained carvacrol (42.65%), thymol (13.38%), γ-terpinene (12.04%), and *p*-cymene (7.17%) as the major phytochemical constituents, classifying it as a carvacrol/thymol/γ-terpinene chemotype [8].

Summer savory (*Satureja hortensis* L.) is an annual herbaceous aromatic plant, belonging to the Lamiaceae family, native to the Mediterranean region, and historically cultivated in the neighboring warm areas of southeastern Europe and southwestern Asia. As cultivation expanded, summer savory spread throughout Europe and is now grown in numerous regions outside its native range. The phenolic compounds identified in summer savory include: phenolic acids (rosmarinic, caffeic, isoferulic, chlorogenic), flavanones (naringenin), flavones (apigenin, apigenin-glucoside, luteolin, vitexin, apigetrin), flavonols (quercetin, isoquercitrin, quercitrin, kaempferol, astragalin), and coumarins (aesculin, aesculetin). Aerial parts of the plant have also been reported to be a rich source of proteins, sugars, fats, fibers, minerals, vitamins, resins, tannins, triterpenic acids, and essential oil. The EO of summer savory comprises terpenes and terpenoids, including monoterpenes (α-terpinene, γ-terpinene, *p*-cymene, α-phellandrene, α- and β-pinene, sabinene, α-thujene, limonene, terpineol, L-carvone), sesquiterpenes (β-bisabolene, caryophyllene oxide), and phenolic monoterpenoids (thymol, carvacrol) [9,10,11]. Similar to thyme, *Satureja* EO displays chemical variability, with different chemotypes determined by the dominance of key constituents and shaped by genotype, environmental and geographical factors [12,13].

Regarding the medicinal applications of summer savory extracts and EO, some secondary metabolites have shown potential as biologically active compounds with antimicrobial, antiseptic, antioxidant, antiparasitic, hepatoprotective, anticancer [14], antiperiodontopathogenic, antibiofilm, regenerative, and immunomodulatory effects [15]. For example, beyond their multiple therapeutic properties, carvacrol and thymol—major constituents of essential oils derived from plants of the Lamiaceae family—have been reported to exhibit pronounced anti-inflammatory activity [16]. Inflammation is a fundamental process involved in the pathogenesis of numerous chronic diseases and can be regulated through the modulation of key molecular signaling pathways, including mitogen-activated protein kinases (MAPKs), nuclear factor-kappa B (NF-κB), tyrosine kinases, and arachidonic acid-related signaling. Available evidence suggests that these compounds act on such pathways, leading to reduced production of pro-inflammatory cytokines and other inflammatory mediators, thereby highlighting their potential as promising pharmaceutical agents [17].

At present, research on essential oils from *Thymus callieri* Borbás ex Velen and *Satureja hortensis* L. has predominantly focused on their phytochemical composition, while data regarding their antioxidant, antimicrobial, anti-inflammatory, and other therapeutic properties remain limited. In particular, in vitro and ex vivo studies on the anti-inflammatory activity of essential oils from thyme (*T. callieri*) and summer savory (*S. hortensis*), especially from Bulgarian populations, have not yet been reported. The selection of the bioassays was based on the interconnected roles of oxidative stress, inflammatory processes, membrane integrity, and microbial growth in the gastrointestinal tract. Therefore, the combined use of antioxidant, antimicrobial, anti-inflammatory, and antihemolytic assays provides a comprehensive assessment of the biological potential of the investigated essential oils. In this context, the present study was designed to investigate their chemical composition and biological activities, with particular emphasis on the ex vivo anti-inflammatory and in vitro antihemolytic effects, thereby providing new experimental evidence for the biological characterization of these aromatic plants.

## 2. Materials and Methods

### 2.1. Materials

#### 2.1.1. Plant Material

**Figure 1:**
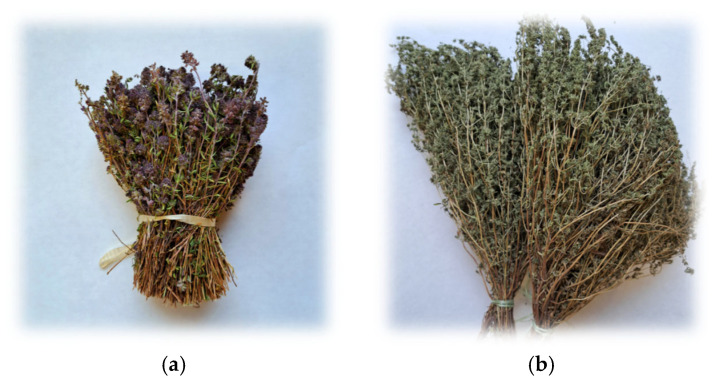
Dried aerial parts of *T. callieri* Borbás ex Velen (**a**) and *Satureja hortensis* L. (**b**) used for obtaining of essential oils. #: Number/No.

Thyme (*Thymus callieri* Borbás ex Velen) (Figure 1a) and summer savory (*Satureja hortensis* L.) (Figure 1b) plant materials were harvested during the flowering period (July–August 2025) and taxonomically identified according to the Herbarium Academiae Scientiarum Bulgariae (Table 1). Following harvest, the aerial parts were shade-dried at 22–25 °C. The leaves and flowers were subsequently manually separated from the stems, which were discarded. The samples were placed in brown paper bags, labeled, and stored at room temperature in darkness until further analyses.

#### 2.1.2. Test Microorganisms

Six Gram-positive bacteria (*Bacillus subtilis* ATCC 6633, *Bacillus cereus* NCTC 11145, *Staphylococcus aureus* ATCC 6538P, *Listeria monocytogenes* NBIMCC 8632, *Enterococcus faecalis* RC-21, *Micrococcus luteus* 2YC-YT), five Gram-negative bacteria (*Salmonella typhimurium* NBIMCC 1672, *Klebsiella pneumoniae* RC-20, *Escherichia coli* ATCC 25922, *Pseudomonas aeruginosa* ATCC 9027, *Proteus vulgaris* ATCC 6380), two yeasts (*Candida albicans* NBIMCC 74, *Saccharomyces cerevisiae*), and four fungi (*Aspergillus niger* ATCC 1015, *Aspergillus flavus*, *Penicillium chrysogenum*, *Fusarium moniliforme* ATCC 38932) from the collection of the Department of Microbiology and Biotechnology at the University of Food Technologies, Plovdiv, Bulgaria, were selected for the antimicrobial activity test.

#### 2.1.3. Culture Media

##### Luria–Bertani Agar Medium with Glucose (LBG Agar)

LBG agar (Laboratorios Conda, S.A., Madrid, Spain) was used for cultivation of test bacteria. A quantity of 50 g of LBG-solid substance mixture (containing 10 g tryptone, 5 g yeast extract, 10 g NaCl, 10 g glucose and 15 g agar) was dissolved in 1 L of deionized water, pH 7.5 ± 0.2.

##### Malt Extract Agar (MEA)

MEA (Scharlab SL, Barcelona, Spain) was used for cultivation of test yeasts and fungi. A quantity of 50 g of the MEA-solid substance mixture (containing 30 g malt extract, 5 g mycological peptone and 15 g agar) was dissolved in 1 L of deionized water, pH 5.4 ± 0.2.

The culture media were prepared according to the manufacturer’s instructions and autoclaved at 121 °C for 20 min (liquid phase) before use.

#### 2.1.4. Animals

Male Wistar rats (*n* = 6) (age of 10–12 weeks) with body weight in the range of 250–280 g were used in the experiment. All animals were housed in the vivarium under standard conditions: living space 350 cm^2^, temperature 22 ± 2 °C, relative humidity 55 ± 10%, and 12/12 h light/dark cycle. The rats (2 to 4 animals per cage) had free access to standard rodent food and water. For the purposes of the experiment, animals were anesthetized with ketamine (90 mg/kg) and xylazine (10 mg/kg) intramuscularly, and then euthanized by cervical decapitation. All animals involved in the experiment were provided by the vivarium of the Medical University of Plovdiv, Bulgaria.

### 2.2. Methods

#### 2.2.1. Isolation of Essential Oils

Plant materials were air-dried at room temperature and ground by a blender to a particle size of 0.5 mm. The dried material was then subjected to hydrodistillation with distilled water for 3 h using a Clevenger-type apparatus to obtain the essential oils. The extracted oils were collected and stored in sealed tubes at 4 °C under refrigeration until further analyses.

#### 2.2.2. Gas Chromatography–Mass Spectrometry (GC–MS) Analysis

The chemical composition of both EOs was analyzed using a gas chromatograph (Agilent Technologies Hewlett Packard 7890A) coupled to a mass spectrometer (Agilent Technologies 5975C inert XL EI/CI MSD, Agilent, Santa Clara, CA, USA), operating at an ionization energy of 70 eV. Prior to analysis, 20 µL of EOs was diluted in 380 µL of hexane. Chromatographic separation was carried out on an HP-5MS capillary column (30 m × 0.32 mm × 0.25 μm) using the following temperature program: initial temperature of 40 °C, increased to 300 °C at a rate of 5 °C/min, with a final hold at 300 °C for 10 min. Helium was used as the carrier gas at a constant flow rate of 1 mL/min. The mass spectrometer was operated in scan mode over a mass range of *m*/*z* 40–400. The injector temperature was set to 250 °C, with a split ratio of 100:1 and an injection volume of 1 μL [18].

#### 2.2.3. Identification of Metabolites

Mass spectral data were processed using AMDIS software version 2.64 (Automated Mass Spectral Deconvolution and Identification System, National Institute of Standards and Technology, NIST, Gaithersburg, MD, USA). Both polar and non-polar compounds were identified by comparing their mass spectra and Kovats’ retention indices (RIs) with reference compounds data from the NIST 08 mass spectral database (NIST Mass Spectral Database, PC Version 5.0, 2008). Retention indices were calculated based on a standard n-hydrocarbon calibration mixture (C10–C40, Fluka) using AMDIS 2.64 software [18].

#### 2.2.4. DPPH Radical Scavenging Assay

The antioxidant capacity of the essential oils was assessed by the DPPH assay according to Ivanov et al. [19], using 2,2-diphenyl-1-picrylhydrazyl (DPPH) reagent (Sigma-Aldrich, St. Louis, MO, USA). The reaction mixture contained 0.15 mL of the tested EO and 2.85 mL of freshly prepared 0.1 mM solution of DPPH in ethanol. The samples were incubated for 30 min in darkness and absorbance was measured at 517 nm. The results were expressed as percentage of inhibition of DPPH radicals, and half-maximal inhibitory concentration (IC_50_). Butylated hydroxytoluene (BHT) was used as a positive control.

#### 2.2.5. Antimicrobial Activity and Minimum Inhibitory Concentration (MIC) Assays

The antimicrobial activity and MIC were evaluated using the agar well diffusion method according to Tumbarski et al. [20]. The essential oils were two-fold serially diluted in methanol to obtain concentrations ranging from 20 mg/mL to 0.0098 mg/mL. Antimicrobial activity was assessed based on the diameter of inhibition zones (IZs) around the wells, measured at 24 and 48 h post-incubation. Microorganisms were classified as sensitive (IZ ≥ 18 mm), moderately sensitive (IZ 12–18 mm), or resistant (IZ ≤ 12 mm or no visible zone). As positive controls, the antibiotics cefotaxime (for bacteria) and nystatin (for yeasts and fungi) were used. Methanol, used as a solvent, served as the negative control.

The MIC values were defined as the lowest concentrations of the essential oils that completely inhibited visible growth of each test microorganism around the agar wells [21].

#### 2.2.6. Ex Vivo Anti-Inflammatory Activity Assay

The ex vivo anti-inflammatory activity assay, including experimental design, induction conditions, and control setup, was performed according to Milusheva et al. [22], with minor modifications described below.

##### Immunohistochemical Analysis

In order to obtain smooth muscle preparations (SMPs), the rats’ stomachs were washed with a physiological serum and cut immediately into stripes (12–13 mm long and 1.0–1.1 mm wide). The SMPs were incubated in 10 mL of Krebs solution containing 20 µL of the tested EO (preliminarily diluted in 1 mL of DMSO) for 1 h. Control samples, including vehicle-treated tissues, were processed under identical experimental conditions. Next, the SMPs were fixed with 10% neutral formalin. After the conventional paraffin wax embedding, serial sections (4 µm thick) were cut to observe the circular and longitudinal layer of the smooth muscle (SM) cells as well as the myenteric plexus of the stomach. They were used for the immunohistochemistry tests.

The sections described above were deparaffinized, and then subjected to the following procedures: detection of antigenic epitopes with citrate buffer, blocking endogenous peroxidase with 3% hydrogen peroxide, blocking endogenous biotin using a kit (ref. No BBK 120, Scy Tek, Lab. Inc., Logan, UT, USA), blocking non-specific binding using a reagent (Superblock, Scy Tek, Lab. Inc., Logan, UT, USA), followed by incubation with interleukin-1β (IL-1β) (E-AB-52153) (Elabscience Biotechnology Inc., Houston, TX, USA), after which a second 10 min incubation followed, with a biotinylated secondary antibody (ref. No AGL015, Scy Tek Lab. Inc., Logan, UT, USA). The reaction was visualized by 3,3′-diaminobenzidine tetrachloride (DAB, Scy Tek Lab. Inc., Logan, UT, USA), and the slices were counterstained with Mayer’s hematoxylin. All microphotographs were taken using a Leica DM1000 LED microscope (Leica Microsystems GmbH, Oxford, UK), combined with Leica ICC50 W digital camera (Leica Microsystems GmbH, Oxford, UK).

##### Morphometric Analysis

The intensity of the immune reaction in the stomach was measured in arbitrary units (AU) on the slices immunostained for IL-1β. Using software, the average intensity of pixels was recorded in arbitrary units in the range 0–256 on microphotographs of the stomach, 0 being black, and 256 being white. A minimum of 50 points were measured in the stomach at magnification ×400. All measurements involved five slices per animal and an examination of all cross-sections of the stomach. The measurements were performed using the LAS X software, version M15.1 (Leica Microsystems GmbH, Oxford, UK).

#### 2.2.7. Red Blood Cell Membrane Stabilization (Antihemolytic Activity)

The red blood cell (RBC) membrane stabilization assay or antihemolytic activity test was performed according to Alamgeer [23] with minor modifications.

##### Preparation of Solutions

Alsever’s solution, hypotonic saline (0.36%), isotonic saline (0.85%), and phosphate-buffered saline (PBS) (pH = 7.2–7.4) were prepared in accordance with the respective formulations [20] and sterilized by autoclaving at 121 °C for 20 min (liquid cycle).

##### Preparation of RBC Suspension

A blood sample was taken from a healthy sheep and gently mixed during the taking with an equal volume of sterilized Alsever’s solution. Thereafter, the blood sample was centrifuged at 3000 rpm for 10 min. The packed blood cells were collected, washed with isotonic saline solution, centrifuged at identical conditions, and then 10% RBC suspension with isosaline was prepared for further analysis.

##### Experimental Procedure

To perform the test, 1 mL of PBS, 2 mL of hypotonic saline, 0.5 mL of EO diluted in DMSO at various concentrations (1, 0.5, 0.25, 0.1 and 0.05 mg/mL) and 0.5 mL of 10% RBC suspension were mixed. As a control, 1 mL of PBS, 2 mL of hypotonic saline, 0.5 mL of DMSO and 0.5 mL of 10% RBC suspension in isosaline was prepared. For the reference controls, 1 mL of PBS, 2 mL of hypotonic saline, 0.5 mL of standard steroid and non-steroidal drug solutions (Prednisolone Cortico and Aspirin) in DMSO at the same concentrations and 0.5 mL of 10% RBC suspension were mixed. Next, the assay mixtures were incubated at 37 °C for 30 min, centrifuged at 3000 rpm for 10 min, the supernatant was transferred to a cuvette and hemoglobin content was measured spectrophotometrically at 560 nm. Percentage protection of RBC against hemolysis was estimated using the following Equation (1):

$$\%\;Protection\;of\;RBC=100-\frac{ODsample}{ODcontrol\;}\times 100$$

The results were also expressed as the half-maximal inhibitory concentration (IC_50_).

#### 2.2.8. Statistical Analysis

The results from triplicate experiments were expressed as mean values ± standard deviation (±SD). One-way analysis of variance (ANOVA) was performed using the Statgraphics Centurion statistical program version XVI, 2009 (Stat Point Technologies, Inc., Warrenton, VA, USA). The mean differences were established by Fisher’s least significant difference test for paired comparison with a significance level of *p* ≤ 0.05. One-sample *t*-test and Wilcoxon test were used for the immunohistochemical analysis. Quantitative data were analyzed using the GraphPad Prism software (GraphPad Software 8.0.1 version, Inc., La Jolla, CA, USA).

## 3. Results and Discussion

### 3.1. GC-MS Analysis of the Chemical Composition of Bulgarian T. callieri and S. hortensis EOs

The identified volatile compounds in the essential oil of *T. callieri* Borbás ex Velen were classified in three main groups with the relevant subgroups as follows: alkylbenzenes, monoterpenes (monoterpene hydrocarbons and oxygenated monoterpenes), and sesquiterpenes (sesquiterpene hydrocarbons). Oxygenated monoterpenes were the predominant group (50.21%), followed by alkylbenzenes (46.42%), monoterpene hydrocarbons (2.7%), and sesquiterpene hydrocarbons (0.48%). A total of 16 chemical compounds were identified in *T. callieri* EO, which are presented in Table 2 in order of their elution from the column. The *T. callieri* EO was classified as the thymol chemotype, with *p*-cymene (46.42%)—known as a biogenetic precursor of thymol—and thymol (35.80%) as the major constituents.

The identified volatile compounds in the essential oil of *S. hortensis* L. were classified in three main groups with the relevant subgroups as follows: alkylbenzenes, monoterpenes (monoterpene hydrocarbons and oxygenated monoterpenes), and sesquiterpenes (sesquiterpene hydrocarbons and oxygenated sesquiterpenes). Oxygenated monoterpenes were the predominant group (59.28%), followed by monoterpene hydrocarbons (26.74%), alkylbenzenes (12.09%), sesquiterpene hydrocarbons (1.32%), and oxygenated sesquiterpenes (0.27%). A total of 17 chemical compounds were identified in *S. hortensis* EO, which are presented in Table 3 in the order of their elution from the column. The major compound detected in *S. hortensis* EO was carvacrol (58.81%), which classifies this EO as the carvacrol chemotype.

To date, a limited number of studies have investigated the chemical composition of *T. callieri* Borbás ex Velen essential oil. A previous study by Rosenova et al. [5], examining *T. callieri* EO from the northeastern Black Sea region of Bulgaria, reported linalool (38.08%), geraniol (27.66%), and geranyl acetate (9.51%) as the major constituents. This composition differed markedly from that of *T. callieri* EO obtained in the present study from the Rhodope Mountains region, as determined by GC–MS analysis. Moreover, the essential oil analyzed in their study lacked *p*-cymene, thymol, and carvacrol, which were identified among the major components in our sample, which could be explained by the different climatic and edaphic conditions in these two geographically distant regions. In another study, Trendafilova et al. [8] investigated *T. callieri* EO from the Struma River Valley and found that carvacrol (42.65%), thymol (13.38%), γ-terpinene (12.04%), and *p*-cymene (7.17%) were the principal components, classifying their essential oil as the carvacrol chemotype. The essential oil of *T. roegneri* (syn. of *T. callieri*) from Turkey was found to contain thymol (56.23%), *p*-cymene (12.94%), and carvacrol (8.59%) as the major chemical constituents, while γ-terpinene was not detected in the sample [24].

The research conducted by Katar et al. [25] to assess variations in the essential oil composition of cultivated summer savory across five different locations in Turkey, showed that the major constituents such as carvacrol ranged from 41.4% to 50.5%, γ-terpinene from 32.7% to 38.7%, and *p*-cymene from 3.4% to 5.5%. These findings demonstrated that the chemical composition of *S. hortensis* EO was influenced by environmental factors specific to each geographical region. According to Rasouli et al. [26], a total of 27 chemical compounds were identified in the EO samples of *S. hortensis*, representing 89.2–99.8% of the total oil. The results indicated that carvacrol (50.84–60.03%) was the dominant component. In addition, γ-terpinene (19.03–27.33%), *p*-cymene (1.73–3.01%), α-terpinene (2.03–2.62%), myrcene (1.28–1.58%), and aromadendrene (1.10–1.52%) were identified as other major constituents of summer savory EO. The major constituents identified by GC-MS analysis in *S. hortensis* essential oil from Iran were carvacrol (42.10%), thymol (19.74%), and *p*-cymene (8.19%) [27].

### 3.2. Antioxidant Activity of T. callieri and S. hortensis EOs

**Figure 2:**
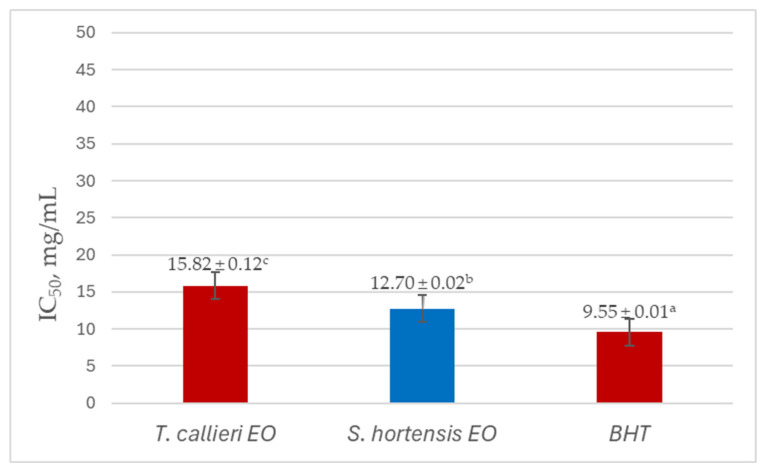
IC_50_ values of *T. callieri* EO, *S. hortensis* EO, and BHT determined by the DPPH method. ^a–c^: Means without a common letter differ significantly (*p* < 0.05).

The free radical scavenging activity of Bulgarian thyme (*T. callieri*) and summer savory (*S. hortensis*) essential oils was measured by DPPH assay. The antioxidant potential of both essential oils and butylated hydroxytoluene (BHT) used as a control is shown in Table 4. The half-maximal inhibitory concentration (IC_50_) values are presented in Figure 2.

The results presented in Table 4 revealed a concentration-dependent antioxidant activity of both *T. callieri* and *S. hortensis* essential oils, as evaluated by the DPPH assay. For both EO samples, the percentage of DPPH inhibition increased with increasing concentration, reaching maximum values (55.38% for thyme EO and 64.61% for summer savory EO) at 20 mg/mL. At all tested concentrations, *S. hortensis* EO exhibited higher radical scavenging activity compared to *T. callieri* EO, indicating comparatively stronger antioxidant potential. Both EOs demonstrated lower antioxidant capacity compared to the positive antioxidant compound BHT.

Overall, the data suggested that *S. hortensis* EO possessed a generally stronger DPPH radical scavenging activity than *T. callieri* EO under the applied experimental conditions, particularly at higher concentrations, which may be attributed to differences in their individual chemical composition and phenolic content.

While antioxidant activity has been reported for extracts of *T. callieri* Borbás ex Velen, the literature currently lacks data on the antioxidant activity of its essential oil. A study investigating the antioxidant potential of the essential oils of *Thymus vulgaris* L. and *Thymus serpyllum* L., collected from central Dalmatia, Croatia, and Bosnia and Herzegovina, demonstrated that the percentage inhibition of DPPH radical scavenging at a concentration of 0.2 g/L was 38.30% and 30.50%, respectively, whereas at a higher concentration of 2 g/L, the values increased to 91.30% and 82.00% [28]. The DPPH radical scavenging activity of *T. vulgaris* essential oil originating from Dammam, Saudi Arabia, ranged from 20.80% at 10 µg/mL to 88.50% at 1000 µg/mL [29].

The antioxidant activity of *S. hortensis* essential oil has been widely reported and is mainly attributed to the high content of terpenes and terpenoids, including carvacrol, *p*-cymene, linalool, thymol, β-caryophyllene, and γ-terpinene, which are considered key contributors to the antioxidant effects. Previous studies have demonstrated significant radical-scavenging capacity using DPPH and ABTS assays, confirming the strong antioxidant potential of *S. hortensis* EO [10]. For instance, Najafian and Zahedifar reported notable DPPH inhibition with IC_50_ values decreasing under optimized cultivation conditions, suggesting that environmental factors can significantly influence antioxidant potential [30]. Other studies have reported that variations in chemical composition and antioxidant activity, particularly increased carvacrol content, are associated with the thermal treatment of samples. In this context, Chambre et al. [31] observed that unheated *S. hortensis* essential oil from Arad, Romania, exhibited 80.02% DPPH inhibition, whereas the heated EO sample showed a higher DPPH inhibition value of 87.73%. A comparative study performed by Elmdoustazar et al. [32] indicated that the essential oil of *S. hortensis* exhibited a similar antioxidant capacity (4.348%) to that of the aqueous extract (4.396%) at a concentration of 50 μg/mL, as determined by the DPPH assay. In their investigation of the antioxidant potential of essential oils obtained from fifteen Iranian *S. hortensis* samples, Samadi et al. [33] reported antioxidant activity index values ranging from 0.17 to 0.46, based on IC_50_ values determined by the DPPH assay. Consequently, the antioxidant capacity of *S. hortensis* EO appears to be strongly dependent on its chemical profile, which is influenced by both genetic, environmental, and other factors.

### 3.3. Antimicrobial Activity of T. callieri and S. hortensis EOs

The antimicrobial activity and minimum inhibitory concentration (MIC) values of thyme and summer savory essential oils, as well as the controls, against various microorganisms are summarized in Table 5.

As seen from the results, the antimicrobial activity of *T. callieri* and *S. hortensis* EOs was evaluated based on their minimum inhibitory concentration (MIC) values. Since lower MIC values indicate higher antimicrobial activity, *T. callieri* EO demonstrated higher antimicrobial potential against some of the test microorganisms. In particular, *T. callieri* EO exhibited the highest inhibitory activity against *B. cereus* (MIC = 0.313 mg/mL), whereas against some other Gram-positive bacteria, including *S. aureus*, *L. monocytogenes*, *E. faecalis*, and *M. luteus*, both essential oils displayed comparable inhibitory effects. *E. faecalis* was the least sensitive strain (MIC = 2.5 mg/mL).

Regarding Gram-negative bacteria, *T. callieri* EO showed the highest antimicrobial activity against *P. aeruginosa* (MIC = 0.313 mg/mL), while on *S. typhimurium* and *E. coli*, both EOs displayed similar inhibitory effects. It is noteworthy that *K. pneumoniae* was among the less susceptible strains, as indicated by the MIC values (2.5 and 1.25 mg/mL, respectively).

Concerning antifungal activity, both EOs showed identical activity against *C. albicans* and *A. niger*. *T. callieri* EO demonstrated stronger inhibitory effect on the fungal strains *A. flavus*, *P. chrysogenum*, and *F. moniliforme*, while *S. hortensis* EO showed higher activity against *S. cerevisiae*. Overall, the results obtained indicated broad-spectrum antimicrobial potential for both essential oils, with *T. callieri* EO exhibiting slightly superior activity in some cases.

It should be noted that the essential oils exhibited antimicrobial activity equal to or greater than that of the conventional antibiotics used as controls against *B. subtilis*, *E. faecalis*, *P. vulgaris*, *C. albicans*, *S. cerevisiae*, *A. niger*, *A. flavus*, *P. chrysogenum*, and *F. moniliforme*. Methanol, which was used as a negative control did not inhibit the growth of the test microorganisms.

The antimicrobial activity of essential oils derived from *Thymus* spp. and *S. hortensis* is closely associated with their ability to disrupt microbial cell structures and interfere with various key physiological processes. These effects are largely attributed to bioactive constituents such as thymol and carvacrol [34,35,36]. Although studies on the antimicrobial activity of *T. callieri* EO are currently unavailable, data from closely related *Thymus vulgaris* suggest a comparable inhibitory potential, largely mediated through disruption of microbial cell membranes and induction of oxidative stress, leading to microbial cell death [37]. In the case of *S. hortensis*, several studies have demonstrated significant inhibitory effects against a broad spectrum of Gram-positive and Gram-negative bacteria, as well as some fungal strains. For instance, Mahboubi and Kazempour [38] reported that *S. hortensis* EO exhibited pronounced antimicrobial activity, largely due to thymol as a major constituent, with MIC values ranging from 0.06 to 16 μL/mL against the tested microorganisms. Similarly, Popovici et al. [39] confirmed the strong inhibitory effect of *S. hortensis* EO, particularly against *S. aureus* ATCC 25923 (inhibition zone = 16 mm), *B. cereus* ATCC 8035 (inhibition zone = 12 mm), and *S. typhimurium* ATCC 14028 (inhibition zone = 11 mm), highlighting the superior antimicrobial efficacy of the EO compared to hydroalcoholic extracts. A study by Tomičić et al. [40] demonstrated the promising antimicrobial activity of *S. hortensis* essential oil from Serbia against three *L. monocytogenes* strains, with MIC values ranging from 256 to 512 µg/mL.

In conclusion, our findings confirmed that the essential oils of *T. callieri* and *S. hortensis* exhibit significant antimicrobial potential, with possible synergistic effects among their constituents, warranting further investigation into their application as natural antimicrobial agents.

### 3.4. Ex Vivo Anti-Inflammatory Activity of T. callieri and S. hortensis EOs

Essential oils (EOs) derived from *Thymus* spp. and *S. hortensis* are well recognized for their biological activities, including antioxidant, anti-inflammatory, and spasmolytic effects. Their major phenolic constituents, such as thymol and carvacrol, play a key role in modulating gastrointestinal smooth muscle function, affecting both muscle contractility and the activity of the enteric nervous system.

Interleukin-1β (IL-1β) is a pro-inflammatory cytokine critically involved in the initiation and maintenance of inflammatory responses in gastrointestinal tissues. Its elevated expression has been associated with altered gastrointestinal motility, including hypermotility, spasms, and enteric neuroinflammation. In this context, the ability of EOs to modulate IL-1β expression suggests their potential as therapeutic agents in functional and inflammatory gastrointestinal disorders.

**Figure 3:**
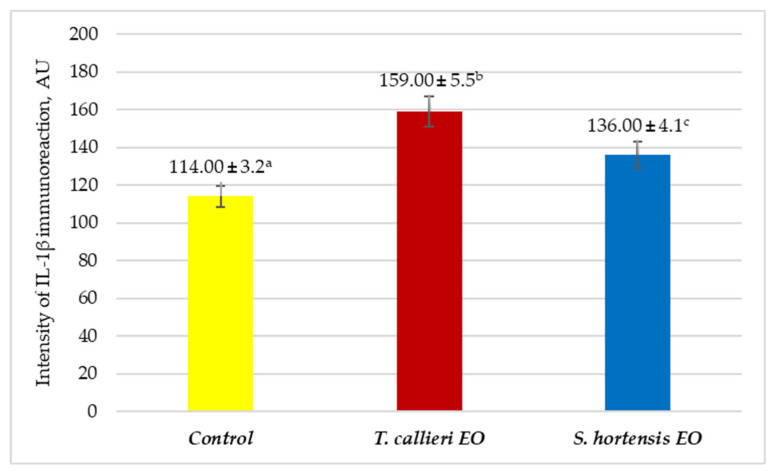
IL-1β expression after treatment with *T. callieri* EO and *S. hortensis* EO (AU). ^a–c^: Means without a common letter differ significantly (*p* < 0.05).

**Figure 4:**
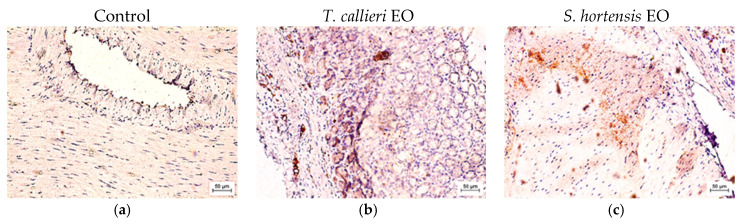
Representative photomicrographs of smooth muscle cells and myenteric ganglia from the rats’ stomach walls: control, stained for IL-1β, ×400 (**a**); sample incubated with *T. callieri* EO (0.18%, *v*/*v*) (**b**); and sample incubated with *S. hortensis* EO (0.18%, *v*/*v*) (**c**), showing the presence of IL-1β expression, ×200.

The activity of interleukin-1β (IL-1β) was assessed using immunohistochemical methods. The results obtained are presented in Figure 3 and Figure 4a–c. Quantitative analysis, expressed in arbitrary units (AU), revealed an increased expression of IL-1β in SMPs treated with *T. callieri* and *S. hortensis* EOs. As shown in Figure 3, the intensity of the immune response was higher in samples incubated with *T. callieri* EO (159.00 ± 5.5 AU) and *S. hortensis* EO (136.00 ± 4.1 AU) compared to the control sample (114.00 ± 3.2 AU). These findings were further supported by the corresponding photomicrographs (Figure 4a–c), which demonstrated enhanced immunoreactivity in the treated SMPs and suggested distinct immunomodulatory effects of the two essential oils on cellular immune responses.

The pronounced increase in IL-1β observed for *T. callieri* EO indicated an elevated pro-inflammatory response in the treated samples. This effect may be associated with its major phenolic constituents (thymol and carvacrol), which are reported to modulate cytokine production and macrophage activity. Although *Thymus* spp. are widely reported to exert anti-inflammatory effects—primarily through inhibition of signaling pathways such as nuclear factor kappa B (NF-κB) and downregulation of pro-inflammatory cytokines as IL-1β, IL-6, tumor necrosis factor α (TNF-α) [41,42]—it can also enhance immune responses under certain conditions, including stimulation of phagocytic activity and nitric oxide (NO) production in macrophages [43]. These findings indicated a context-dependent modulation of inflammatory signaling pathways. In contrast, *S. hortensis* EO exhibited a lower, moderate increase in IL-1β expression, corresponding to higher ex vivo anti-inflammatory activity. These results were consistent with the earlier reported anti-inflammatory and immunoregulatory properties of summer savory EO. Previous studies have shown that *S. hortensis* can suppress LPS-induced production of IL-1β and other pro-inflammatory cytokines in macrophages [44], likely through modulation of NF-κB signaling and antioxidant mechanisms [42]. The presence of phenolic compounds such as carvacrol contributes to the attenuation of oxidative stress and downstream inflammatory cascades [9]. Additionally, in vivo data indicated that *S. hortensis* can influence both humoral and cellular immune responses, supporting a balanced immunomodulatory profile rather than strong immune activation [45].

Taken together, these findings suggest that *T. callieri* EO exerted a more pronounced stimulatory effect on innate immune responses, as reflected by higher IL-1β expression, whereas *S. hortensis* EO demonstrated a moderate anti-inflammatory activity. These differences are likely attributable to variations in phytochemical composition and their interaction with key inflammatory pathways. Consequently, *T. callieri* may be more suitable in contexts requiring immune activation, while *S. hortensis* may offer benefits in conditions associated with excessive or chronic inflammation.

### 3.5. In Vitro Antihemolytic Activity (Red Blood Cell Membrane Stabilization) of T. callieri and S. hortensis EOs

The in vitro antihemolytic activity of *T. callieri* and *S. hortensis* essential oils was evaluated using sheep erythrocytes as an experimental model and compared with the commercial steroid (Prednisolone Cortico) and non-steroidal (Aspirin) anti-inflammatory drugs used as controls at equivalent concentrations.

**Figure 5:**
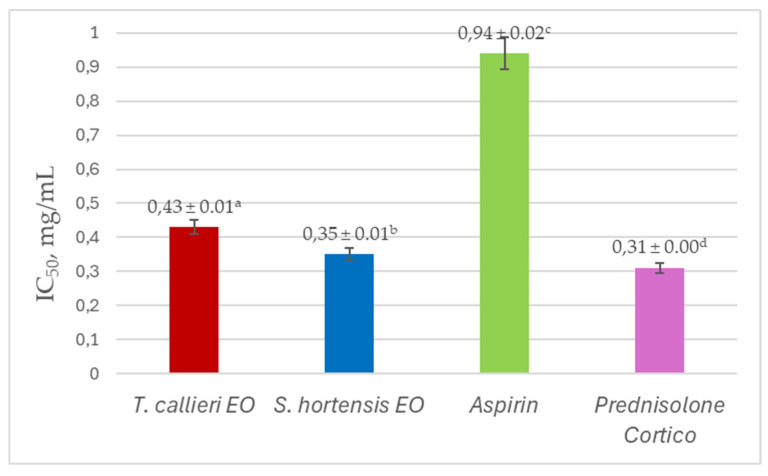
IC_50_ values of *T. callieri* and *S. hortensis* essential oils, and reference anti-inflammatory drugs in the in vitro antihemolytic assay. ^a–d^: Means without a common letter differ significantly (*p* < 0.05).

As seen from the results in Table 6, at 1 mg/mL no protective effect was observed for either EO, whereas Aspirin, and especially Prednisolone Cortico showed remarkable antihemolytic activity (51.61% and 93.77%, respectively). It is noteworthy that *S. hortensis* EO demonstrated greater protection from hypotonicity-induced hemolysis, compared to *T. callieri* EO, and exceeded the effect of Aspirin. A concentration-dependent decrease in erythrocyte protection was observed at lower concentrations (0.25, 0.1, and 0.05 mg/mL) for all tested samples. The reference steroid anti-inflammatory drug Prednisolone Cortico showed the strongest antihemolytic effect at all tested concentrations. Regarding the individual IC_50_ values, they ranged between 0.31 mg/mL (Prednisolone Cortico) and 0.94 mg/mL (Aspirin) (Figure 5).

Essential oils obtained from *S. hortensis* L. seeds have been reported to exhibit significant anti-inflammatory activity, as demonstrated in in vivo models. Experimental studies, particularly those employing carrageenan-induced paw edema, have shown that the EO effectively reduced inflammatory responses, often accompanied by notable analgesic effects [46]. These biological activities are largely attributed to the high content of monoterpenes such as γ-terpinene, thymol, and carvacrol, which are known to modulate key inflammatory pathways [10]. In contrast, specific data on the anti-inflammatory potential of *T. callieri* Borbás ex Velen have not yet been reported. However, some studies on other *Thymus* spp., particularly *T. vulgaris*, have demonstrated pronounced anti-inflammatory effects, including the inhibition of pro-inflammatory mediators and reduction in oxidative stress in cellular systems [47]. Given the similarity in chemical composition among *Thymus* species, it is reasonable to assume that *T. callieri* EO may exhibit comparable biological activity, which further underlines the importance of its investigation.

The erythrocyte membrane is structurally similar to the lysosomal membrane, and its stabilization suggests that T. callieri and S. hortensis EOs may exert a stabilizing effect on lysosomal membranes. Maintaining lysosomal membrane integrity is a crucial factor for limiting inflammatory processes, as it prevents the release of lysosomal enzymes from activated neutrophils, which would otherwise exacerbate inflammation and contribute to tissue damage. Most anti-inflammatory drugs act either by stabilizing cellular membranes or by inhibiting hydrolytic enzymes. Therefore, inhibition of erythrocyte hemolysis is commonly used as an indicator of anti-inflammatory activity due to the structural resemblance between erythrocyte and lysosomal membranes [48].

Although direct evidences for anti-hemolytic (red blood cell membrane stabilization) activity of *T. callieri* and *S. hortensis* essential oils are currently lacking, their antioxidant properties and membrane-interacting effects have been widely described. The major monoterpenes in these oils such as thymol and carvacrol are known potent antioxidants that contribute to membrane stability and protection against oxidative stress damage—mechanisms indirectly associated with anti-hemolytic activity [49]. Furthermore, some plant extracts obtained from members of the Asteraceae family have exhibited membrane-stabilizing effects on rat erythrocyte model, supporting the relevance of this type of anti-inflammatory activity evaluation [50].

## 4. Conclusions

To the best of our knowledge, this study provides new data on the essential oil of *Thymus callieri* Borbás ex Velen, including its antioxidant, antimicrobial, anti-inflammatory, and anti-hemolytic potential, as well as the ex vivo anti-inflammatory and antihemolytic activities of the essential oil of *Satureja hortensis* L., representing a novel contribution to the field. The results demonstrated that the analyzed essential oils exhibited a diverse composition of bioactive compounds and significant biological potential. The strong antioxidant, antimicrobial, and anti-inflammatory effects can be attributed to the major constituents thymol and carvacrol. The obtained results were in agreement with previously reported data, while in some cases indicating enhanced bioactivity, thus confirming the high biological potential of these essential oils. Based on these findings, thyme and summer savory essential oils can be considered promising natural sources for various applications in the pharmaceutical, food, and cosmetic industries. Future research should be focused on optimizing extraction techniques, as well as evaluating the effects of cultivation conditions and harvest periods on the yield and bioactive profile of the essential oils to maximize their practical applications.

## Figures and Tables

**Table 1:** Origin of thyme (*T. callieri* Borbás ex Velen) and summer savory (*S. hortensis* L.) samples used in the study.

Sample	Village/Town	District/Region	GPS Coordinates	Altitude, m	Voucher #
Thyme	Near Dospat	Smolyan	41°66′ N 24°16′ E	1214	SOM 176971
Summer savory	Krichim	Plovdiv	42°04′ N 24°46′ E	253	SOM 179942

**Table 2:** Phytochemical profile of thyme (*T. callieri* Borbás ex Velen) essential oil by GC-MS analysis.

Peak	RT	RI	Name	% of TIC
Alkylbenzenes				
5	12.32	1021	*p*-Cymene	46.42 ± 1.67
Monoterpenes				
*Monoterpene hydrocarbons*				
1	9.20	934	α-Pinene	1.49 ± 0.05
2	9.70	946	Camphene	0.33 ± 0.01
3	10.61	971	β-Pinene	0.09 ± 0.00
4	11.07	987	β-Myrcene	0.26 ± 0.02
6	12.38	1023	Eucalyptol	0.34 ± 0.03
7	12.45	1025	Limonene	0.19 ± 0.01
*Oxygenated monoterpenes*				
8	14.67	1095	β-Linalool	2.72 ± 0.25
9	16.11	1143	β-Terpineol	0.32 ± 0.03
10	16.50	1160	Isoborneol	0.14 ± 0.02
11	16.78	1164	Borneol	0.39 ± 0.07
12	17.54	1189	α-Terpineol	3.73 ± 0.46
13	17.61	1198	γ-Terpineol	3.26 ± 0.29
14	20.43	1292	Thymol	35.80 ± 1.35
15	20.60	1300	Carvacrol	3.85 ± 0.18
Sesquiterpenes				
*Sesquiterpene hydrocarbons*				
16	23.60	1418	β-Caryophyllene	0.48 ± 0.05
Total identified, %				99.81

RT—retention time; RI—retention index; TIC—total ion current.

**Table 3:** Phytochemical profile of summer savory (*S. hortensis* L.) essential oil by GC-MS analysis.

Peak	RT	RI	Name	% of TIC
Alkylbenzenes				
7	12.24	1021	*p*-Cymene	12.09 ± 0.53
Monoterpenes				
*Monoterpene hydrocarbons*				
1	9.00	917	Tricyclene	0.77 ± 0.06
2	9.22	929	α-Pinene	1.01 ± 0.02
3	10.64	974	β-Pinene	0.23 ± 0.01
4	11.09	991	β-Myrcene	0.74 ± 0.03
5	11.60	1004	α-Phellandrene	0.10 ± 0.00
6	11.99	1010	α-Terpinene	1.13 ± 0.16
8	12.38	1024	Limonene	0.19 ± 0.02
9	12.44	1027	Eucalyptol	0.11 ± 0.00
10	13.28	1060	γ-Terpinene	22.46 ± 0.89
*Oxygenated monoterpenes*				
11	14.64	1096	β-Linalool	0.21 ± 0.04
12	17.22	1156	Terpinen-4-ol	0.16 ± 0.01
13	20.16	1304	Carvacrol	58.81 ± 1.95
14	22.25	1373	Carvacryl acetate	0.10 ± 0.00
Sesquiterpenes				
*Sesquiterpene hydrocarbons*				
15	23.69	1420	β-Caryophyllene	0.41 ± 0.07
16	25.81	1545	β-Bisabolene	0.91 ± 0.14
*Oxygenated sesquiterpenes*				
17	27.61	1587	Caryophyllene oxide	0.27 ± 0.09
Total identified, %				99.70

RT—retention time; RI—retention index; TIC—total ion current.

**Table 4:** Antioxidant activity of thyme (*T. callieri*) EO, summer savory (*S. hortensis*) EO, and butylated hydroxytoluene (BHT) determined by the DPPH method.

Concentration, mg/mL	Inhibition, %		
	*T. callieri*	*S. hortensis*	BHT
20	55.38 ± 0.23 ^a^	64.61 ± 0.53 ^a^	79.18 ± 0.89 ^a^
10	43.09 ± 0.76 ^b^	49.67 ± 0.76 ^b^	53.03 ± 1.15 ^b^
5	30.26 ± 0.64 ^c^	35.35 ± 0.69 ^c^	44.49 ± 0.78 ^c^
2.5	21.98 ± 0.68 ^d^	24.96 ± 0.58 ^d^	28.81 ± 2.88 ^d^
1.25	13.87 ± 0.64 ^e^	16.80 ± 0.34 ^e^	23.82 ± 2.88 ^d^
0.625	10.80 ± 0.59 ^f^	12.38 ± 0.53 ^f^	16.01 ± 2.46 ^e^

^a–f^: Means in a column without a common letter differ significantly (*p* < 0.05); BHT—Butylated hydroxytoluene.

**Table 5:** Antimicrobial activity and minimum inhibitory concentration (MIC) values of essential oils from thyme (*T. callieri*) and summer savory (*S. hortensis*) and controls.

Test Microorganisms	MIC, mg/mL				
	Essential Oils		Controls		
	*T. callieri*	*S. hortensis*	Cefotaxime	Nystatin	MeOH
*Bacillus subtilis* ATCC 6633	0.625	1.25	0.625	n.a.	-
*Bacillus cereus* NCTC 11145	0.313	0.625	0.0197	n.a.	-
*Staphylococcus aureus* ATCC 6538P	0.625	0.625	0.0393	n.a.	-
*Listeria monocytogenes* NBIMCC 8632	1.25	1.25	0.079	n.a.	-
*Enterococcus faecalis* RC-21	2.5	2.5	2.5	n.a.	-
*Micrococcus luteus* 2YC-YT	1.25	1.25	0.079	n.a.	-
*Salmonella typhimurium* NBIMCC 1672	0.625	0.625	0.0393	n.a.	-
*Klebsiella pneumoniae* RC-20	2.5	1.25	0.0197	n.a.	-
*Escherichia coli* ATCC 25922	0.625	0.625	0.0393	n.a.	-
*Proteus vulgaris* ATCC 6380	0.625	1.25	2.5	n.a.	-
*Pseudomonas aeruginosa* ATCC 9027	0.313	0.625	0.0393	n.a.	-
*Candida albicans* NBIMCC 74	1.25	1.25	n.a.	2.5	-
*Saccharomyces cerevisiae* ATCC 9763	1.25	0.625	n.a.	1.25	-
*Aspergillus niger* ATCC 1015	1.25	1.25	n.a.	2.5	-
*Aspergillus flavus*	0.313	1.25	n.a.	2.5	-
*Penicillium chrysogenum*	0.625	1.25	n.a.	5	-
*Fusarium moniliforme* ATCC 38932	1.25	2.5	n.a.	5	-

n.a.—not applied.

**Table 6:** In vitro antihemolytic activity of thyme (*T. callieri*) and summer savory (*S. hortensis*) EOs.

Sample	Protection, %				
	1 mg/mL	0.5 mg/mL	0.25 mg/mL	0.1 mg/mL	0.05 mg/mL
*T. callieri* EO	-	54.08 ± 0.23 ^a^	40.88 ± 0.43 ^b^	13.20 ± 0.92 ^c^	8.10 ± 0.84 ^d^
*S. hortensis* EO	-	67.31 ± 1.20 ^a^	43.68 ± 0.29 ^b^	26.64 ± 0.76 ^c^	20.45 ± 0.22 ^d^
Aspirin	51.61 ± 0.33 ^a^	47.31 ± 0.55 ^b^	30.27 ± 0.37 ^c^	13.67 ± 0.12 ^d^	9.43 ± 0.07 ^e^
Prednisolone	93.77 ± 1.85 ^a^	75.27 ± 0.83 ^b^	47.98 ± 0.32 ^c^	37.41 ± 0.78 ^d^	30.03 ± 0.17 ^e^

^a–e^: Means in a row without a common letter differ significantly (*p* < 0.05).

## Data Availability

Datasets from the time of this study are available from the respective authors upon reasonable request.
